# A Computationally Efficient and Accurate Method for Predicting Conductance of Single-Molecule Junctions

**DOI:** 10.1021/acs.nanolett.6c01462

**Published:** 2026-06-01

**Authors:** Artem Gulyaev, Jyotisman Hazarika, Zhen-Fei Liu, Latha Venkataraman

**Affiliations:** † 148492 Institute of Science and Technology Austria, 3400 Klosterneuburg, Austria; ‡ Department of Chemistry, 2954 Wayne State University, Detroit, Michigan 48202, United States; § Department of Applied Physics and Department of Chemistry, Columbia University, New York, New York 10027, United States

**Keywords:** Density Functional Theory, Single-Molecule Conductance, Electron Transport, Transmission Function

## Abstract

Despite significant progress in the field of molecular electronics over the last two decades, the quantitative prediction of metal-molecule-metal junction conductance remains a challenge. The standard computational framework combines density functional theory (DFT) with nonequilibrium Green’s functions (NEGF) using low-rung exchange-correlation functionals such as PBE, which overestimate the conductances. More advanced correction methods exist but require complex workflows and high computational cost, limiting their accessibility. Here, we introduce a physically motivated approach that approximates results obtained with high-rung functionals. Our method fits the PBE-calculated transmission to a Breit-Wigner form and subsequently refines the fit parameters using molecular orbital energies and metal densities of states computed for the isolated subsystems with high-rung functionals. This approach is applicable to a broad range of molecular junctions yielding conductance values in quantitative agreement with experiments. Our approach is simple, low-cost, and accurate, making it well-suited for routine and large-scale prediction of single-molecule junction conductance.

## Graphical abstract

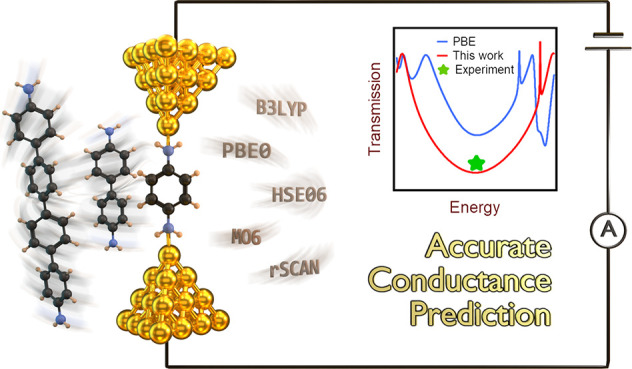

Electron transport measurements of single-molecule devices have come a long way since the early 2000s.^1–3^ Reproducible measurements of single-molecule junction conductances are now routine, especially utilizing the scanning tunnelling microscope-based break-junction (STM-BJ) technique under ambient conditions.^4–6^ Over the same period, computational chemistry has seen major progress;^7,8^ however, accurately modeling single-molecule junctions from first-principles remains challenging, in both capturing various junction geometries^9–12^ and accurately describing the electronic structure,^13–16^ and a fully quantitative comparison between measured and calculated conductance has yet to be achieved. Density functional theory (DFT) with functionals that better capture electron exchange and correlation, such as hybrid functionals like B3LYP or PBE0, have improved performance in isolated molecular properties compared to low-rung functionals such as the generalized gradient approximations (GGAs).^17^ However, when a molecule is bound to metal electrodes, determining the energy-dependent transmission function for the combined system with high-rung functionals^18–21^ is computationally expensive. Calculations using low-rung functionals, such as the Perdew–Burke–Ernzerhof (PBE) of the GGA rung, place transmission resonances too close to the metal Fermi energy, leading to conductance values that are overestimated in the linear-response regime.^22,23^ Importantly though, the difference between calculations using PBE and those using high-rung functionals or beyond-DFT approaches is largely systematic and therefore amenable to correction. One such correction scheme is the DFT+ Σ method,^22,23^ which corrects resonance positions by accounting for both the molecular self-interaction error and a surface polarization effect. Although this method improves conductance estimates significantly, it requires nontrivial, multistep workflows, making it less accessible. Moreover, it does not explicitly correct the molecule-metal coupling described by a low-rung functional.

Here, we present an approximate method to predict the conductance of single-molecule devices, benchmark it against DFT-NEGF calculations that use high-rung functionals, and demonstrate that it accurately agrees with experimental results for a broad range of molecules. The method consists of fitting a PBE-calculated energy-dependent transmission function *T*(*E*) to the Breit-Wigner form and adjusting the fit parameters through a physically motivated process that yields a more accurate transmission function.^24–26^ We apply this method to a series of aromatic molecules and compare our calculations with experimentally obtained junction conductance data. We find that the low-bias conductance as well as the conductance decay parameter β agrees well with our experiments. Furthermore, we emphasize that this approach can be applied to correct transmission functions calculated from any low-rung functionals (i.e., the starting point does not need to be PBE) using any computational packages. Our method therefore provides an accurate, accessible, and molecule-unspecific approach for bridging the gap between theory and experiment.

We first present calculations for a prototypical molecular junction formed using Au electrodes and 1,4-benzenediamine (BDA) where the nitrogen lone pair forms a donor–acceptor bond to undercoordinated gold atoms (Figure 1a). The conductance of this molecule has been experimentally measured and found to be around 7 × 10^–3^ G_0_.^23,27^ We calculate the energy-dependent transmission of a molecular junction formed with BDA bound to two Au electrodes. For these calculations, we first perform a geometry relaxation of the molecule with two Au atoms attached (one on each N) using the PBE^28^ functional as implemented in the FHI-aims software package.^29,30^ We then append Au_21_ pyramidal electrodes to each Au atom to form a molecular junction as shown in Figure 1a and compute the transmission using the AITRANSS package.^8,31,32^ The transmission function (Figure 1a) shows multiple resonances that reach unity within the energy window shown. The three resonances that dominate transmission near the Fermi energy (*E*_F_) are indicated by the arrows in Figure 1a. These are derived from the HOMO, LUMO, and the LUMO+1, as can be seen in Figure 1b, which compares the orbitals of the isolated molecule and those of the molecular junction. Transmission at *E*_F_ is dominated by the HOMO and LUMO+1 and overestimates the experimental conductance by a factor of 5.

**Figure 1:**
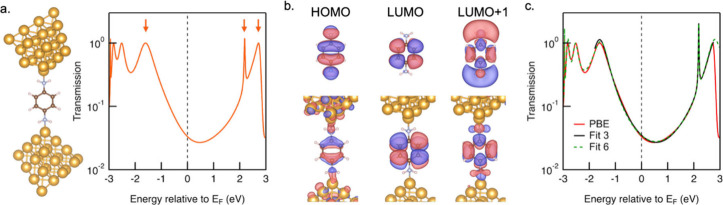
a. Structure of BDA bound to two pyramidal gold electrodes and calculated transmission function versus energy using the PBE functional. Arrows indicate the dominant resonances that dictate transmission at *E*_F_. b. Orbital isosurface plots for the isolated molecule (top) and those obtained from the junction at the energies indicated by the arrows in panel a (bottom). Isosurface value of 0.05 was used for the plots. c. Fits of eq 1 to the PBE transmission function using 3 resonances (solid black) and 6 resonances (dashed green).

To develop a practical procedure to improve the theoretical prediction, we fit the PBE transmission function with the Breit-Wigner formula (eq 1).^33^ For a symmetric molecule, each peak bears three independent fit parameters: resonance energy *ε*_*n*_, peak width Γ_*n*_, and phase θ_*n*_. We note here that for asymmetric molecules, or molecules with frontier resonances that do not reach unit transmission, we would need to include two coupling parameters (Γ_*n*,*R*_ and Γ_*n*,*L*_) for each resonance. By adjusting the fitting method, one can make the fit achieve greater accuracy near the peaks or the valleys. However, to capture the experimentally measured conductance, it is important to respect both; to this end, we do a least-squares fit of the ln­(*T*(*E*)) to the natural logarithm of eq 1. Our fitting code is provided as a Supporting Information (SI) along with the fit parameters. We show in Figure 1c two fits, one fitting the region that includes three resonances (HOMO, LUMO, and LUMO+1) and one fitting six resonances (HOMO–2 to LUMO+2). In principle, any number of resonances can be included, but the principle of parsimony dictates that we use the minimal number of resonances needed to capture the transmission around *E*_F_ well—three, in this case.

1 $$T\left(E\right)={\left|\underset{n}{\sum }e^{i\theta_{n}}\frac{\Gamma_{n}}{E-\varepsilon_{n}+i\Gamma_{n}}\right|}^{2}$$

Having obtained the fit parameters, we attempt to correct the well-documented self-interaction error that low-rung functionals make.^8,32^ Specifically, high-rung functionals predict lower energies than PBE for occupied MOs and higher energies for unoccupied ones.^34^ To accommodate this, the energy of each resonance is shifted by the energy difference between PBE and a high-rung functional, calculated for a corresponding orbital of an isolated molecule. The correction via eq 2 is similar to the gas-phase term in the DFT+ Σ method.^35^

2 $$\varepsilon_{n}^{\left(\mathrm{func},\mathrm{derived}\right)}=\varepsilon_{n}^{\left(\mathrm{PBE},\mathrm{fit}\right)}+\varepsilon_{n}^{\left(\mathrm{func.,isol.}\right)}-\varepsilon_{n}^{\left(\mathrm{PBE},\mathrm{isol}\right)}$$

We apply this correction using two hybrid functionals, B3LYP and PBE0. B3LYP was chosen for its popularity, while PBE0 was chosen because the calculated ionization potential with PBE0 is the closest to the experimental one. *ε*_*n*_^(func, derived)^ is then substituted into eq 1 to produce an improved *T*(*E*).

To understand if this shift actually leads to a *T*(*E*) that agrees with a full calculation made using B3LYP and PBE0 using the geometries optimized with the PBE functional, we compare in Figure 2a the transmission and fit results. While the peak positions are indeed correctly predicted to within a few percent, the corrected transmissions deviate from those calculated using hybrid functionals in the region of interest near *E*_F_. This difference, often exceeding a factor of 2, persists for all functionals. Although the transmission at *E*_F_ is now closer to the experimental value, it is clear that correcting only the resonance positions does not reproduce the results from a full calculation.

**Figure 2:**
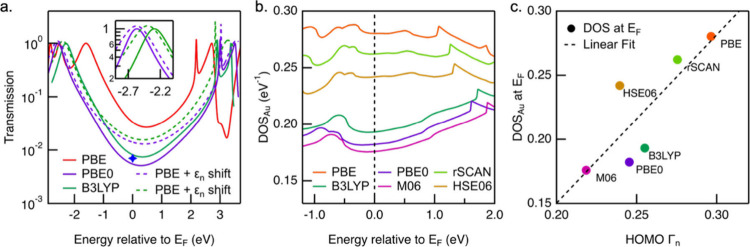
a. Transmission function versus energy for a molecular junction formed with BDA bound to two pyramidal Au electrodes calculated using PBE (red), PBE0 (purple), and B3LYP (green). Dashed lines are derived by combining gas-phase orbital energy shift (eq 2) and the 3 resonance fits (eq 1) to the latter two functionals. The experimental value is indicated with a star. b. DOS of face-centered cubic bulk Au calculated using a series of functionals indicated in the legend. c. DOS_Au_ at *E*_F_ versus Γ_*n*_ for HOMO from fits of transmission functions shown in panel a and Figure S1. A linear fit to the data is also shown.

Evidently, other fit parameters must also be tuned to address this discrepancy. Since the shape and nodal structure of MOs remain largely unchanged between functionals, we can conclude that the phase parameters θ_*n*_ should not be changed. However, we see in Figure 2a that the resonances from the corrected transmissions are wider than those of the direct calculation using high-rung functionals. We therefore need to tune the Γ_*n*_ parameters to capture the differences in the coupling of the molecule to the leads. In beyond-DFT approaches such as DFT+∑, this phenomenological coupling parameter is also implicitly altered,^36^ after a self-energy correction is made to the molecular subspace. Generally, the coupling strength Γ_*n*_ is energy dependent and is proportional to the electrode’s density of states [DOS_Au_(*E*)],^25^ i.e.,

3 $$\Gamma \left(E\right)={\left|\tau \left(E\right)\right|}^{2}\mathrm{DO}S_{\mathrm{Au}}\left(E\right)$$

where τ­(*E*) is the state-to-state hopping parameter between the MO and the Au states at energy *E*. Assuming that τ­(*E*) is approximately constant or similar for different functionals, we scale the fitting parameter Γ_*n*_ by the ratio of the DOS_Au_ calculated using the high-rung functional and PBE.

To test this hypothesis, we calculate the transmission of the BDA junction following the procedure detailed above using five other functionals (hybrids: B3LYP, PBE0, HSE06; meta-GGAs: M06, rSCAN). All calculated transmission functions are shown in Figure S1. We then fit these with eq 1 to determine *ε*_*n*_, Γ_*n*_, and θ_*n*_ for each of the three resonances. Additionally, we calculate, using FHI-Aims, the DOS of bulk face-centered cubic Au using the same six functionals (shown in Figure 2b) and a 20 × 20 × 20 k-mesh. We find that the fitting parameter Γ_*n*_ for the HOMO scale roughly with the DOS_Au_ at *E*_F_ across the six functionals as shown in Figure 2c. We thus correct the fitted eq 1 (from PBE) by scaling Γ_*n*_ for each resonance as follows:

4 $$\Gamma_{n}^{\left(\mathrm{func},\mathrm{derived}\right)}=\Gamma_{n}^{\left(\mathrm{PBE},\mathrm{fit}\right)}\cdot \frac{\mathrm{DO}S_{\mathrm{Au}}^{\left(\mathrm{func}\right)}\left(E_{F}\right)}{\mathrm{DO}S_{\mathrm{Au}}^{\left(\mathrm{PBE}\right)}\left(E_{F}\right)}$$

Note that the scale factor above is molecule independent and is easily transferable for different junctions. We now compare the transmission for a BDA junction calculated using B3LYP and PBE0 with that obtained from PBE using the scaled Γ_*n*_ (eq 4) and shifted *ε*_*n*_ (eq 2) in Figure 3a. Data for the other functionals considered here are shown in SI Figure S1. In all cases, the agreement is more quantitative than those obtained without scaling Γ_n_’s (shown in Figure 2a). Importantly, the PBE0-predicted conductance at zero bias is 5.5 × 10^–3^ *G*_0_, while that from the fit-and-correct procedure is 5.4 × 10^–3^ *G*_0_ and both are in reasonable agreement with the experimental value of 7.0 × 10^–3^ *G*_0_, measured at 100 mV (SI Figure S4).

**Figure 3:**
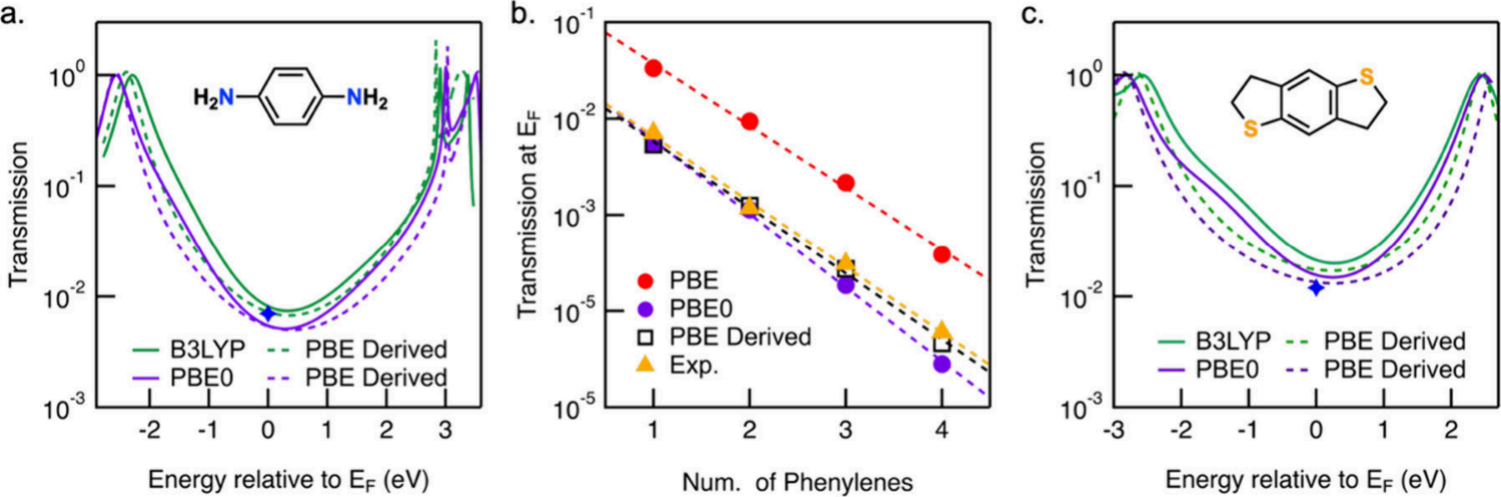
a. Transmission function versus energy for a BDA junction calculated using PBE0 (solid purple) and B3LYP (solid green) along PBE-derived transmissions after applying corrections following eqs 2 and 4, where the corrections are based on PBE0 (dashed purple) or B3LYP (dashed green). The experimental value is indicated with a star. b. Transmission for a series of oligophenylenediamines with 1–4 phenyls compared with the experimental conductance determined from data collected at a 100 mV bias. c. Transmission versus energy for a thiomethyl-terminated benzene calculated with PBE0 (purple) and B3LYP (green) along PBE-derived transmissions after applying corrections following eqs 2 and 4. The experimental value is indicated with a star.

To test this procedure for a range of molecules, we first consider a series of oligophenylenediamines with 1–4 phenylene units and carry out calculations using PBE0 and B3LYP. In each case, we calculate the transmission of the junction using PBE and the hybrid functional. We then fit the PBE transmission with eq 1 using either two or three resonances and correct the fit parameters as described by eqs 2 and 4 to obtain a PBE-derived transmission. The results from this process are shown in Figure 3b, where we apply PBE0-based corrections. We compare these results to the experimental ones, where the most probable conductance is determined from fits to the linear-binned histogram as opposed to a logarithm-binned histogram, as the latter skews conductance values toward higher conductances. Our experimental conductances are in excellent agreement with what our approach predicts.

We next test the procedure for a molecule terminated by thiomethyl linkers. Here, we choose a locked-thiomethyl linker (see inset of Figure 3c) due to its reduced spread in experimentally obtained conductances. We again compare the B3LYP and PBE0 calculations with the PBE-derived transmission by applying the corrections following eqs 2 and 4. As shown in Figure 3c, applying these corrections to the PBE transmission results in a very good approximation to the full calculation using high-rung functionals. Additionally, the experimental conductance 1.2 × 10^–2^ G_0_ matches closely the transmission at *E*_F,_ 1.3 × 10^–2^ G_0,_ for the PBE-derived transmission with PBE0-based correction. A comparison of the experimental and calculated values for all molecules considered here is provided in Table 1. We also apply the same methods to a meta-coupled molecule 3,3′-biphenyl diamine (33DBDA, Figure S5) and demonstrate that the theory works well even for molecules that evidence destructive quantum interference in the transmission. Note that the transmission at *E*_F_ is below our instrument noise floor at low biases, and therefore we cannot compare this with an experimental value.

**Table 1:** Comparison of the Experimental Most Probable Conductance Determined from a Linearly Binned Conductance Histogram Shown in SI Figure S4 along with the Calculated Conductance Values^a^

molecule	experiment	direct PBE0 calculation	PBE-derived fit with PBE0-based correction
BDA	7.0 × 10^–3^	5.5 × 10^–3^	5.4 × 10^–3^
DBDA	1.2 × 10^–3^	1.1 × 10^–3^	1.2 × 10^–3^
TBDA	3.1 × 10^–4^	1.8 × 10^–4^	2.7 × 10^–4^
TetBDA	6.0 × 10^–5^	2.8 × 10^–5^	4.6 × 10^–5^
SMePh	1.2 × 10^–2^	1.6 × 10^–2^	1.3 × 10^–2^

a All conductance values are given in the units of conductance quantum *G*_0_.

Finally, we repeat our calculations of BDA but using Ag electrodes. For these calculations, we followed the same procedure as described above with Au electrodes. We calculate the transmission of a BDA molecule bound to two Ag_22_ clusters using six different functionals (PBE, B3LYP, PBE0, M06, rSCAN, and HSE06) and compare these with the transmission determined using the PBE functional after correcting it using eqs 2 and 4 above based on isolated molecule and DOS calculation with the respective functionals. We show our results in Figure 4 with additional details presented in Figure S6. We compare the results to the experimental value from a previously published work.^37^ The agreement between the experiment and theory is again very good.

**Figure 4:**
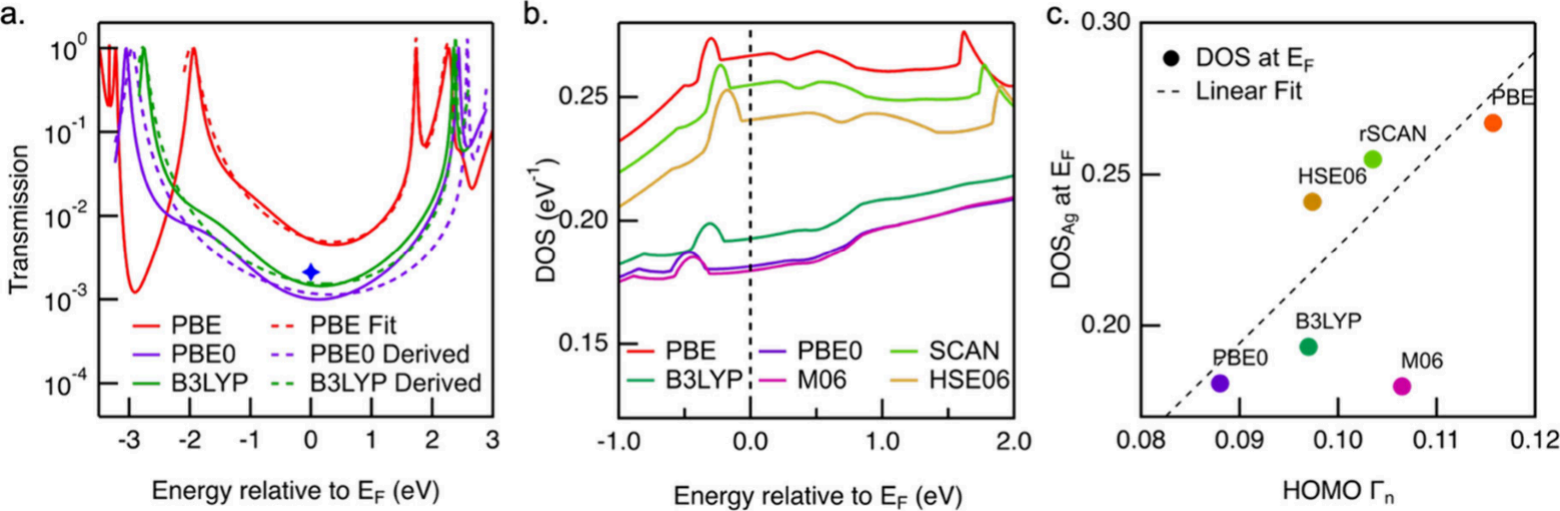
a. Transmission function versus energy for a molecular junction formed with BDA bound to two pyramidal Ag electrodes calculated using PBE (red), PBE0 (purple), and B3LYP (green). Dashed lines are the PBE-derived transmissions after applying corrections following eqs 2 and 4, where the corrections are based on PBE0 (dashed purple) or B3LYP (dashed green). The star indicates the experimental value for this molecular junction based on the work of Kim et al.^37^ b. DOS of face-centered cubic bulk Ag calculated using a series of functionals indicated in the legend. c. DOS_Ag_ at *E*_F_ versus Γ_*n*_ for HOMO from fits of transmission functions shown in panel a and SI Figure 5. A linear fit to the data excluding M06 is also shown.

To conclude, we developed a computationally efficient method to closely approximate high-rung DFT calculations of single-molecule junction transmission functions and benchmarked these against direct high-rung calculations and experiment. We showed that applying solely an energy shift to resonance energies from a PBE calculation was insufficient for reproduction of high-level calculations, but scaling the resonance widths according to the ratio of the DOS of bulk metal at the Fermi level calculated with high-rung functionals allowed us to make the approximation more quantitative. The framework described here provides an accessible, low-cost, physically motivated, and molecule-unspecific method to emulate high-level calculations and predict experimental low-bias conductances, applicable for a broad range of molecules and functionals. There are of course some limitations to our method. (1) For junctions with organometallic molecules such as metal porphyrins,^38^ the ordering of molecular orbitals may depend on the functional, and thus a simple shift of PBE-based calculations may not always yield correct results. (2) The Breit-Wigner formula explicitly assumes two contacts and cannot capture the transmission through molecular junctions that are bound to electrodes with multiple anchoring groups.^39^ (3) The extension of this approach to self-assembled monolayers requires further work, where intermolecular interactions alter the single-molecule picture.^40^ (4) The extension of this approach to finite bias requires further work, where the evolutions of both molecular resonance energy^41^ and molecule-electrode coupling^42^ with bias need to be properly taken into account.

## Supplementary Material





## Notes

Transmission calculation details, transmission curve fitting details, conductance data, molecular geometries (PDF)
Fitting code (TXT)
