# Supplementary material for: A Computationally Efficient and Accurate Method for Predicting Conductance of Single-Molecule Junctions
Source: Nano Lett. 2026 Jun 1;26(22):7429–34. doi: 10.1021/acs.nanolett.6c01462 (PMC13267173; doi:10.1021/acs.nanolett.6c01462)
Supplement: Supplementary file 1 [file nl6c01462_si_001.pdf]

Supplementary Information for:

A Computationally Efficient and Accurate Method for Predicting Conductance of Single-Molecule Junctions

Artem Gulyaev[1], Jyotisman Hazarika[1], Zhen-Fei Liu[2], Latha Venkataraman[1,3]

[1]Institute of Science and Technology Austria, 3400 Klosterneuburg, Austria

[2]Department of Chemistry, Wayne State University, Detroit, Michigan 48202, USA

[3]Department of Applied Physics and Department of Chemistry, Columbia University, New York, New York 10027, USA

Contents:

1. Transmission Calculation Details
2. Transmission Curve Fitting Details
3. Conductance Data
4. Additional Data
5. Fitting Code
6. Molecular Geometries
7. References

1. Transmission Calculation Details

1.1 Complete transmission calculation procedure

All the calculations have been performed in FHI-aims,[1, 2] with default 'tight' basis for all atoms except Au and 'light' basis for Au. The standard geometry relaxation threshold of 5e-3 eV/Å was used. The atomic ZORA scalar correction was used for relativistic corrections. The transmission functions were calculated with the AITRANSS package.[3, 4]

For each molecule, transmission calculations followed these steps:

1) Geometry optimization was performed for an isolated molecule.
2) Two Au atoms were attached to the geometry output of Step 1, one to each linker. Geometry was optimized using the standard criteria.
3) Three additional layers of Au (111) (with the unit cell parameter being 4.07 Å for the face-centred cubic gold) were attached to each side, forming a pyramid. Two additional atoms were attached to each of the two pyramids at asymmetric locations to break the junction symmetry. A total of 22 Au atoms were used on each side to mimic the Au electrode.
4) AITRANSS calculations are performed on this geometry with all the DFT functionals mentioned in the main text.
5) The output geometry of Step 2 was taken, both gold atoms removed, and the single-point calculations with a DFT functional of interest were performed to find the orbitals and their energies for the isolated molecule.

Optimized molecular geometries are provided at the end of this document.

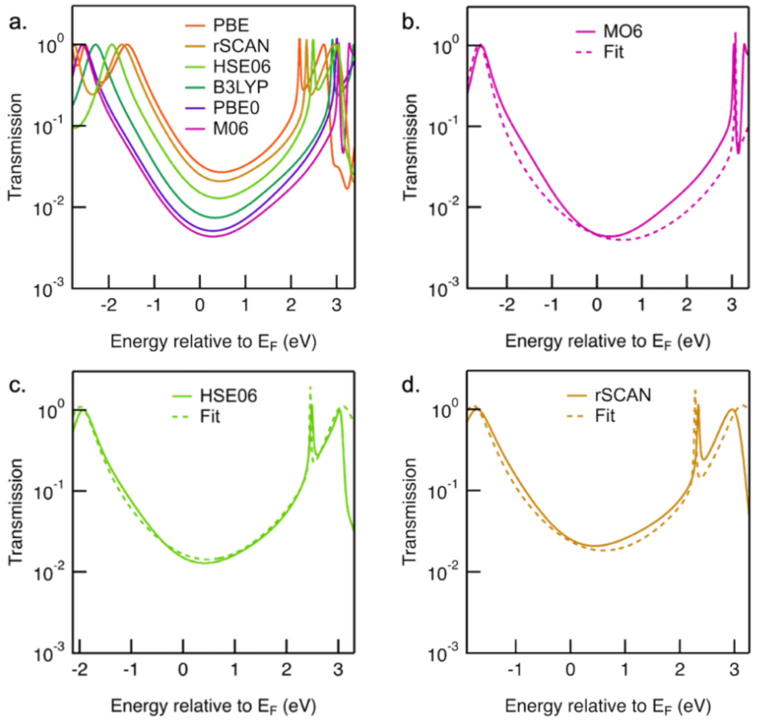

Figure S1. a. Transmission function of Au-BDA-Au junctions calculated with all functionals considered here along with PBE-derived transmission by applying corrections using Eqs. 2 and 3 of the manuscript for three resonance fits using b. MO6, c. HSE06 and d. rSCAN.

1.2 Isolated molecular orbital energies

Name	Orbital number	Orbital type	PBE, eV	B3LYP, eV	PBE0, eV
BDA	29	HOMO	-4.046	-4.843	-5.019
	30	LUMO	-0.905	-0.254	-0.058
	31	LUMO+1	-0.346	0.079	0.368
DBDA	49	HOMO	-4.324	-5.013	-5.193
	50	LUMO	-1.232	-0.627	-0.463
TBDA	69	HOMO	-4.503	-5.154	-5.340
	70	LUMO	-1.711	-1.127	-0.990
TetBDA	89	HOMO	-4.623	-5.258	-5.449
	90	LUMO	-1.963	-1.386	-1.262
SMePh	51	HOMO	-4.389	-5.128	-5.312
	52	LUMO	-1.350	-0.708	-0.540
33DBDA	48	HOMO-1	-5.000	-5.840	-6.040
	49	HOMO	-4.920	-5.700	-5.900
	50	LUMO	-1.600	-0.980	-0.820

The orbital energies of BDA from three other functionals are listed below. The functional M06 changed the order of antibonding orbitals, introducing N-H antibonding orbitals, which contribute little to conductance. The precise correspondence can be checked via isosurface plots or scalar products of PBE and M06 orbitals, which must be close to 1 for the corresponding ones.

Name	Orbital number	Orbital type	rSCAN, eV	HSE06, eV	M06, eV
BDA	29	HOMO	-4.226	-4.431	-5.066
	30 (33 for M06)	LUMO	-0.810	-0.631	-0.012
	31 (36 for M06)	LUMO+1	-0.054	-0.113	0.751

1.3 Density of states calculation

Gold: The density of states of gold was calculated with FHI-aims on a face-centred cubic lattice with lattice parameter 4.07 Å, exactly as in the electrodes. The primitive unit cell containing 1 Au atom was used. A 20 x 20 x 20 k-mesh was used for all calculations. The precise geometry of the unit cell was:

```
lattice_vector  0.000000  2.039000  2.039000
lattice_vector  2.039000  0.000000  2.039000
lattice_vector  2.039000  2.039000  0.000000
atom_frac  0.0  0.0  0.0  Au
```

The values of density of states at the Fermi level are given below:

Functional	DOS at Fermi level (arbitrary unit)	Ratio to PBE DOS
PBE	0.280	1
B3LYP	0.193	0.689
PBE0	0.182	0.650
rSCAN	0.262	0.935
HSE06	0.242	0.863
M06	0.176	0.628

Silver: The density of states of gold was calculated with FHI-aims on a face-centred cubic lattice with lattice parameter 4.07 Å, exactly as in the electrodes. The primitive unit cell containing 1 Ag atom was used. A 20 x 20 x 20 k-mesh was used for all calculations. The precise geometry of the unit cell was:

```
lattice_vector  0.000000  2.039000  2.039000
lattice_vector  2.039000  0.000000  2.039000
lattice_vector  2.039000  2.039000  0.000000
atom_frac  0.0  0.0  0.0  Ag
```

The values of density of states at the Fermi level are given below:

Functional	DOS at Fermi level (arbitrary unit)	Ratio to PBE DOS
PBE	0.266	1
B3LYP	0.193	0.726
PBE0	0.181	0.680
rSCAN	0.255	0.959
HSE06	0.241	0.906
M06	0.180	0.677

2. Transmission Curve Fitting Details

The exact code for fitting transmission curves with Eq. 1 in the manuscript is provided with detailed documentation as a supplementary code file. Some key aspects of fitting transmission data are provided here. As mentioned in the manuscript, in doing the fit, it is important that both resonances and the valleys between the resonances of the transmission function are fit well. The resonance energies, ε_n, have to be approximately aligned with the maxima of the transmission function, otherwise applying energy shifts will lead to inaccuracies. The valleys should also be fit well, because in most cases, conductance measurements are in the off-resonance regime, and thus the region between the HOMO and LUMO resonances is the most important to get quantitative agreement with experiment.

The fitting algorithm works by first providing some starting parameters for the ε_n, Γ_n and θ_n values. These are obtained from the PBE transmission by noting the location of the resonances, their approximate full width at half maximum, and using a phase of either 0 or π. Once the algorithm is run, the output parameters must be examined to ensure that they are physically reasonable. For example, if the code converges on negative values of Γ_n, which is possible if too many resonances are being fit or initial guesses are unreasonable, one should reduce the number of resonances being fit.

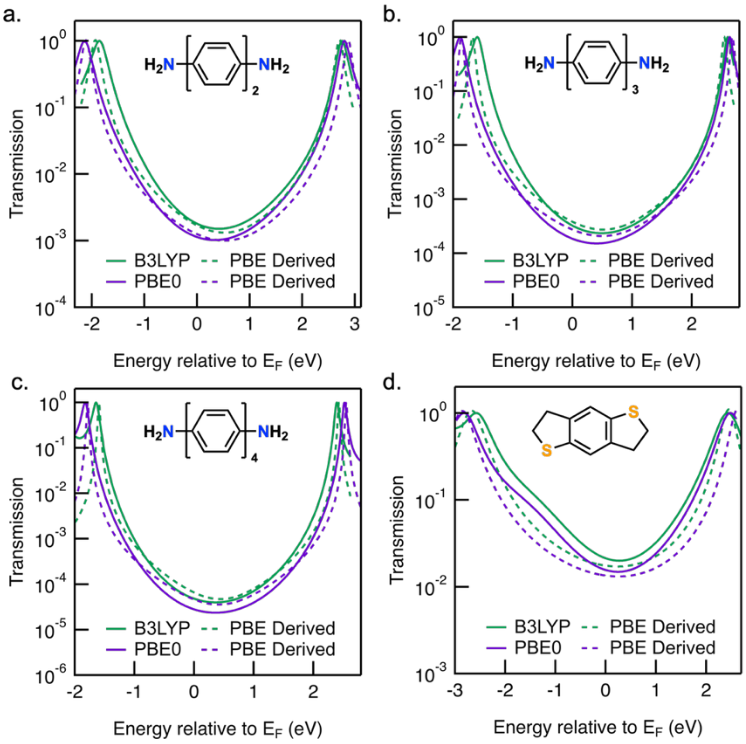

Figure S2. PBE-based transmission calculations for the oligophenylenediamine series with 2-4 phenylenes and for the SMe-terminated phenyl group along with the fit of Eq. 1 for each transmission fitting just the HOMO and LUMO resonances. This minimal fit was sufficient to capture the transmission curve around E_F.

We used the algorithm to fit the data for all the molecules given in the text. The fit parameters for each molecule and the resulting plots are given below. The phase θ_n is the phase of the second (also third for BDA) peak relative to the first.

Name	Energy range, eV	Resonance energies ε_n relative to E$_F$, eV	Resonance widths Γ_n, eV	Phases θ_n, rad
BDA	(-1.79, 2.75)	-1.595, 2.185, 2.845	0.296, 0.011, 0.315	0.122, 1.043
DBDA	(-1.36, 2.17)	-1.241, 2.101	0.135, 0.117	0.939
TBDA	(-1.22, 2.04)	-1.025, 1.961	0.0601, 0.0549	0.875
TetBDA	(-1.11, 2.02)	-0.957, 1.851	0.0261, 0.0254	0.780
SMePh	(-2.15, 2.07)	-1.932, 1.812	0.377, 0.241	1.682
33DBDA	(-2.54, 2.08)	-1.99, -1.68, 1.95	0.085, 0.061, 0.013	3.134, 0.538
BDA-Ag	(-2.10, 2.26)	-1.98, 1.73, 2.25	0.116, 0.017, 0.074	0.610, 1.864

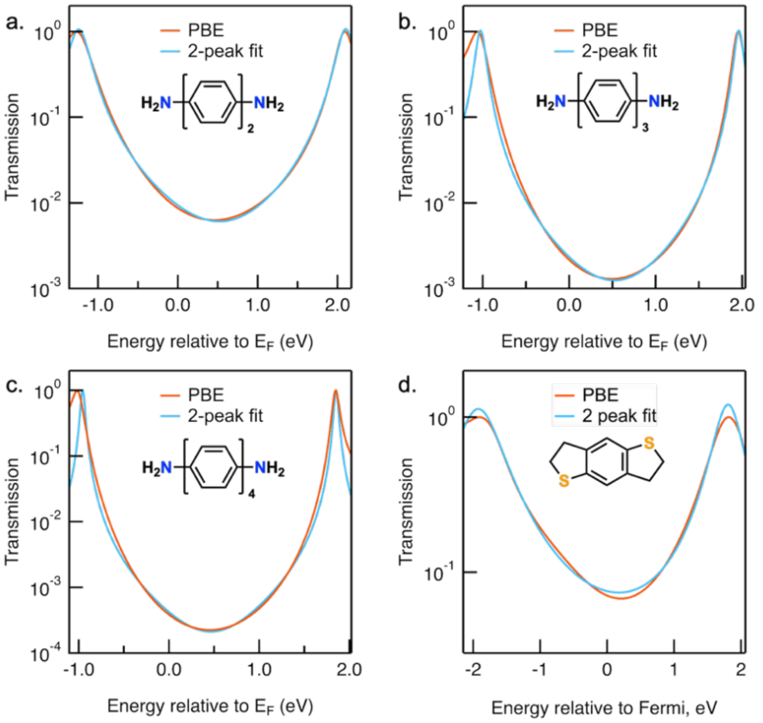

Figure S3. B3LYP and PBE0 transmission calculations for the oligophenylenediamine series with 2-4 phenylenes and SMePh along with PBE-derived transmission by applying corrections using Eqs. 2 and 3. In all cases, the fits reproduce the trends of the calculations with the high-rung functionals.

In general, it is possible to make the peak widths Γ_n depend on energy. Using the formula[5] $\Gamma(E) = |\tau(E)|^2 \mathrm{DOS}(E)$ and assuming approximately constant molecule-electrode coupling $\tau(E)$, the most reasonable energy dependence is the following:

$$\Gamma_\mathrm{n}(E) = \frac{\mathrm{DOS_{Au}}(E)}{\mathrm{DOS_{Au}}(\varepsilon_n)} \cdot \Gamma(\varepsilon_n)$$

Since the gold DOS depends only weakly on energy in the vicinity of the Fermi level, the thus-produced $T(E)$ curve differs only slightly from the constant-Γ_n curve shown in the main text. We therefore do not include an energy-dependent $\Gamma(\mathrm{E})$ in our analysis.

3. Conductance Data

Scanning tunnelling microscope-based break-junction measurements were carried out using a custom instrument as detailed previously.[6] All molecules considered for this work were obtained commercially as reported in the original publications.[6, 7] TetBDA was measured at low biases specifically for this work and obtained from Sigma Aldrich and used without further purification.

Conductance data used in this work are reproduced below.

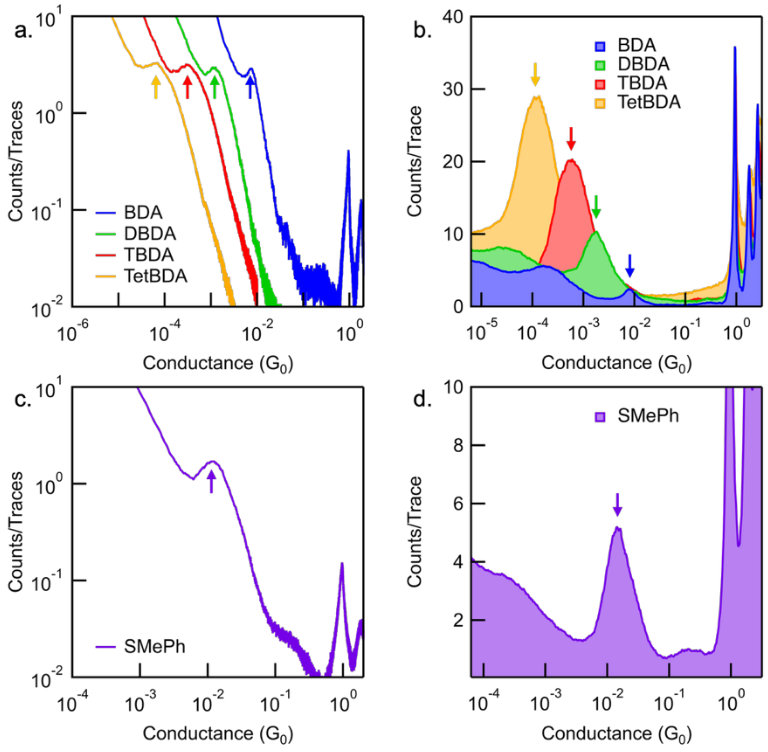

Figure S4. Conductance histograms for oligophenylenediamine series and for SMePh showing both linear binned data (a,c) and log binned data (b,d). Conductance values were taken from linear binned histograms as these values are not skewed towards higher conductances.

4. Additional Data

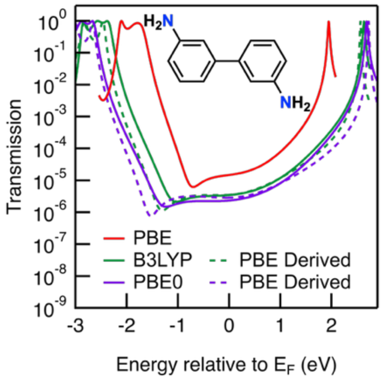

Figure S5. PBE, B3LYP and PBE0 transmission calculations for the para-coupled biphenyldiamine (3,3'-biphenyldiamine) PBE-derived transmission by applying corrections using Eqs. 2 and 3.

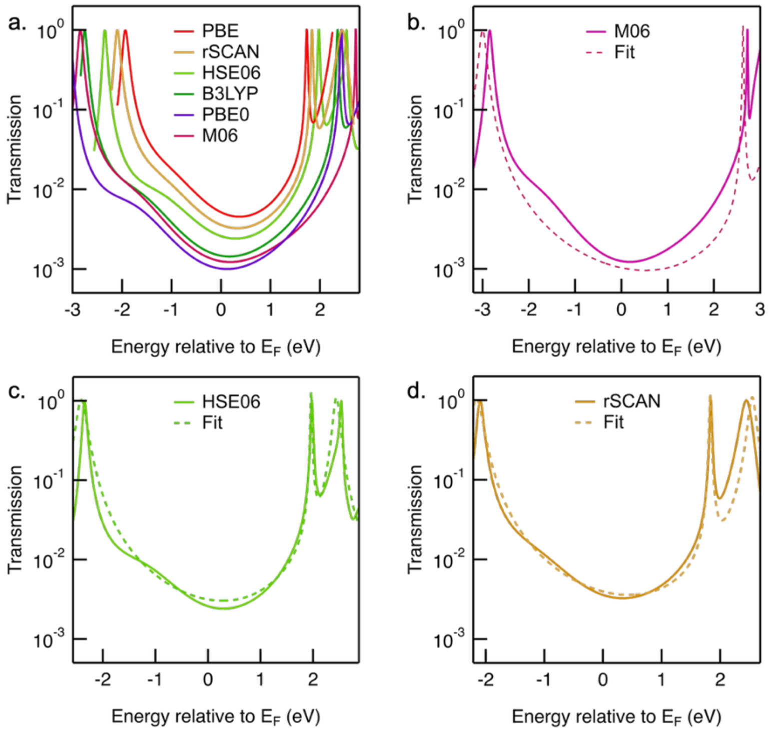

Figure S6. Transmission function of Ag-BDA-Ag junctions calculated with all functionals considered here along with PBE-derived transmission by applying corrections using Eqs. 2 and 3 of the manuscript for three resonance fits using b. MO6, c. HSE06 and d. rSCAN.

4. Fitting Code

The fitting python code is provided as a supplemental file. The steps are as follows:

1. Read the DFT PBE based transmission versus energy data as two arrays (E_PBE, T_PBE).

2. Identify the region within this loaded data that needs to be fit.

3. Estimate the peak positions and resonances that need to be fit.

4. Generate the model transmission using the estimated peak positions and widths using Eq. 1 in the manuscript on a log scale and perform a least-squares fit to the data tuning the parameters.

5. Molecular Geometries

Below are the optimized geometries of each molecule with 2 Au atoms.

BDA (n = 1)

C	-3.77154207	1.84530727	0.18459363
C	-3.06018840	2.83462762	-0.52139237
C	-1.68451639	2.75912928	-0.64167483
C	-0.97671419	1.67543377	-0.08700216
C	-1.68799344	0.68629302	0.61922318
C	-3.06372081	0.76182743	0.73958044
H	-3.59875840	3.66440240	-0.98056967
H	-1.14321408	3.54684363	-1.16708138
H	-1.14946936	-0.14352375	1.07833564
H	-3.60493114	-0.02543640	1.26565559
N	-5.16544567	1.88694031	0.24887534
N	0.41727857	1.63369191	-0.15184603
H	-5.58165685	2.80871175	0.14669534
H	0.83540781	2.14823012	-0.92247296
H	-5.58425931	1.37209384	1.01885235
H	0.83304515	0.71160330	-0.05073492
Au	1.82445064	2.65281082	1.52955535
Au	-6.57073204	0.86820366	-1.43467252

DBDA (n = 2)

C	-7.99343915	3.86705173	0.09477662
C	-7.26151210	2.69996981	0.37272408
C	-7.29591547	5.05871824	-0.16685870
C	-5.91081883	5.06891034	-0.18276264
C	-5.87656816	2.72110394	0.34931845
C	-5.15466988	3.90239295	0.06815995
H	-7.85021085	5.97626436	-0.36972872
H	-5.40023330	5.99887796	-0.43059679
H	-7.78884344	1.77844425	0.62404793
H	-5.33774284	1.80473002	0.58811281
C	-3.68928658	3.91609576	0.04185819
C	-2.94589259	2.75377213	-0.26127193
C	-2.95459822	5.09213851	0.31166894
C	-1.56966528	5.10869524	0.29145127
C	-1.56086171	2.75951235	-0.28908765
C	-0.85039862	3.93555913	0.00613542
N	0.54770094	3.95879954	-0.06742494
H	1.00521217	3.06227290	0.07569724
H	0.99842959	4.69615444	0.46814996
N	-9.39199043	3.86149674	0.16149668
H	-9.83076650	2.95869766	0.00046354
H	-9.85439460	4.59899823	-0.36376892

H	-3.46777676	1.83160084	-0.51474355
H	-1.01691578	1.85277056	-0.55805484
H	-3.48201684	6.00769979	0.57728812
H	-1.03208623	6.03307354	0.50820646
Au	1.69825821	4.45465866	-2.14267209
Au	-10.56461677	4.29715040	2.23844511

TBDA (n = 3)

C	-8.73843556	-0.74140763	0.03717602
C	-8.10792246	0.50029112	-0.14128360
C	-6.72356860	0.58650603	-0.14446654
C	-7.94694821	-1.88328452	0.23863783
C	-6.56324036	-1.78507672	0.22905359
C	-5.90949109	-0.55073511	0.03599534
H	-8.71090028	1.39811170	-0.28543709
H	-6.25978261	1.55659519	-0.32122211
H	-8.42435538	-2.84762179	0.41935365
H	-5.97308165	-2.68349521	0.40796530
C	-4.44203839	-0.45328662	0.03179333
C	-3.78040671	0.71725682	0.45390093
C	-3.63328704	-1.52719195	-0.39109555
C	-2.24822754	-1.43686645	-0.39083970
C	-2.39543199	0.80731222	0.45470124
C	-1.58645497	-0.26714314	0.03357657
H	-4.10135484	-2.43681250	-0.76790436
H	-4.36285128	1.55766006	0.83178181
H	-1.92771646	1.71668106	0.83239665
H	-1.66599649	-2.27739068	-0.76879155
C	-0.11887784	-0.17154599	0.03926608
C	0.53787129	1.06405567	-0.13559267
C	0.69272495	-1.31089135	0.21741505
C	2.07704223	-1.22511361	0.23187775
C	1.92152068	1.16195287	-0.12688146
C	2.71005180	0.01811433	0.07478782
H	0.22718968	-2.28265724	0.37952037
H	2.67807301	-2.12439011	0.37532169
H	-0.04945912	1.96455412	-0.31310392
H	2.40141080	2.12779509	-0.29232261
N	4.11200370	0.10441247	0.04301698
H	4.49129632	1.01461343	0.29222644
H	4.59764162	-0.64259523	0.53333247
H	-10.63417239	-0.08857542	-0.40228353
H	-10.52219873	-1.74188205	-0.13665850
N	-10.13962354	-0.82758818	0.09118307
Au	-11.32170221	-0.56926250	2.19076464
Au	5.32410964	-0.12357818	-2.04041145

TetBDA (n = 4)

C	-5.00818808	0.39541047	-0.02579440
C	-4.34530726	1.59353652	-0.35397382

C	-4.20581573	-0.71315572	0.30552601
C	-2.95861950	1.67879665	-0.34805784
C	-2.15685644	0.57049833	-0.01615425
C	-2.81910691	-0.62814938	0.30940116
C	-0.68536882	0.66120992	-0.00790762
C	-6.47902128	0.30549221	-0.02776989
C	-7.28292517	1.41821926	0.28899579
C	-8.66875776	1.33833422	0.29292924
C	-9.30700215	0.12758879	-0.01601943
C	-8.52507388	-0.98775775	-0.35265335
C	-7.13987019	-0.89628569	-0.35003211
H	-6.81051885	2.36022337	0.56669759
H	-9.26528454	2.21663894	0.54438228
H	-9.00962097	-1.92422986	-0.63318961
H	-6.55536886	-1.77091713	-0.63480833
H	-4.92713360	2.46355189	-0.65920001
H	-2.48427319	2.61397744	-0.64652537
H	-2.23587137	-1.49829753	0.61159521
H	-4.67891719	-1.64843572	0.60580869
C	-0.02620758	1.85714662	0.33345910
C	0.11955219	-0.44519040	-0.33849075
C	1.36050974	1.94130861	0.34681051
C	1.50622490	-0.36069133	-0.32736146
C	2.16607851	0.83454340	0.01708549
H	-0.61227603	2.72567652	0.63481965
H	1.83035506	2.87368365	0.66069271
H	-0.35191031	-1.37794675	-0.64888460
H	2.09113128	-1.22942538	-0.63051457
C	3.63695729	0.92226531	0.03452065
C	4.30498530	2.12664866	-0.26228706
C	5.69026746	2.21433694	-0.24709992
C	6.46585156	1.08639432	0.06134537
C	5.81967803	-0.11837013	0.37714800
C	4.43372792	-0.19435225	0.35566889
H	3.72622062	3.00772716	-0.53868972
H	6.17986630	3.16052474	-0.48268664
H	3.95535239	-1.13576239	0.62509200
H	6.40973959	-0.99260963	0.65647944
N	7.86876956	1.18093844	0.12501830
H	8.27168143	1.92308220	-0.44196974
H	8.36177924	0.30297541	-0.01848845
N	-10.71197027	0.05276980	-0.05958593
H	-11.19297897	0.73707644	0.51924603
H	-11.09394810	-0.87897356	0.08361075
Au	-11.90293729	0.53086325	-2.11286284
Au	8.96777193	1.78215112	2.19890485

SMePh

C	-6.06922814	1.97696144	0.25933593
C	-5.44829158	3.17448955	0.55784095

C	-5.30016963	0.86557600	-0.17530531
C	-3.90355725	0.96244802	-0.35812862
C	-4.05151153	3.27107613	0.37566153
C	-3.28258201	2.15998752	-0.05937746
H	-6.02491023	4.03174158	0.90581004
H	-3.32722611	0.10575853	-0.70796764
C	-1.80274191	2.44553520	-0.07046232
C	-1.65065261	3.96630148	-0.13873220
H	-1.28353507	1.95544162	-0.90517946
H	-1.35489324	2.07724632	0.86788060
S	-3.08705272	4.65217169	0.78413156
H	-1.73514864	4.34524845	-1.16585126
H	-0.73660573	4.34722708	0.32871093
C	-7.54897941	1.69171341	0.26947209
C	-7.70156844	0.17108492	0.33861650
H	-7.99588739	2.05885562	-0.66963789
H	-8.06879542	2.18262161	1.10322700
S	-6.26478252	-0.51525802	-0.58395716
H	-8.61538336	-0.20967778	-0.12936347
H	-7.61718563	-0.20764141	1.36576305
Au	-2.15913162	4.65828287	3.02276717
Au	-7.19384981	-0.52006186	-2.82216456

33DBDA

C	-7.96036200	3.93529900	-0.10463100
C	-7.24826200	4.93953300	-0.75436400
C	-7.26239100	2.93272000	0.58291200
C	-5.86280100	2.93701700	0.59146600
C	-5.85382000	4.95386500	-0.73474200
C	-5.14168000	3.94572100	-0.06105700
H	-7.78960300	5.71752900	-1.29238500
H	-5.31271900	5.73077800	-1.27332200
C	-3.65979400	3.93868400	-0.03947700
C	-2.94216200	2.73056000	-0.09761300
C	-2.94519100	5.14147500	0.03855800
C	-1.54515300	5.14643700	0.05287500
C	-1.54779900	2.73771600	-0.07375800
C	-0.84167700	3.93463900	0.00702500
H	-3.47805200	1.78682200	-0.19143700
H	-1.00163200	1.79606400	-0.12651800
H	-3.48350600	6.08652600	0.12497300
N	-0.84603600	6.36673600	0.19092700
H	-9.05055100	3.92169400	0.13174600
H	0.24846800	3.93449300	0.04058900
H	-5.32861500	2.15559700	1.13397700
N	-7.96721000	1.88573500	1.21895600
H	-8.91464100	2.12829400	1.49974100
H	-7.46437300	1.44881500	1.98833200
H	-1.34712800	7.18055900	-0.15800100

H	0.10548000	6.34725500	-0.16878400
Au	-8.46695600	-0.11930400	-0.02360200
Au	-0.34580400	7.21208100	2.39872900

BDA/Ag

C	-3.77668200	1.84851600	0.19408400
C	-3.06913200	2.81383900	-0.53924600
C	-1.68869500	2.73259600	-0.67393300
C	-0.97234100	1.67295300	-0.09584800
C	-1.67978900	0.70834600	0.63843000
C	-3.06019500	0.78970400	0.77335800
H	-3.60697700	3.63588000	-1.01409700
H	-1.15720000	3.50534100	-1.23162300
H	-1.14152500	-0.11394200	1.11251000
H	-3.59166100	0.01800100	1.33244400
N	-5.18448800	1.88458500	0.25979100
N	0.43520600	1.63517500	-0.16406000
Ag	-6.68814900	0.73517900	-1.49716700
H	-5.58074000	2.81629700	0.16561900
Ag	1.94869400	2.78269500	1.58824200
H	0.82414600	2.09976300	-0.98067000
H	-5.57420000	1.41900400	1.07545200
H	0.83076800	0.70325800	-0.06936500

6. References

(1) Blum, V.; Gehrke, R.; Hanke, F.; Havu, P.; Havu, V.; Ren, X.; Reuter, K.; Scheffler, M., Ab initio molecular simulations with numeric atom-centered orbitals, *Comp. Phys. Commun.,* **2009,** 180, 2175–2196.

(2) Havu, V.; Blum, V.; Havu, P.; Scheffler, M., Efficient integration for all-electron electronic structure calculation using numeric basis functions, *J. Comp. Phys.,* **2009,** 228, 8367–8379.

(3) Bagrets, A., Spin-Polarized Electron Transport Across Metal–Organic Molecules: A Density Functional Theory Approach, *J. Chem. Theory Comput.,* **2013,** 9, 2801–2815.

(4) Arnold, A.; Weigend, F.; Evers, F., Quantum chemistry calculations for molecules coupled to reservoirs: Formalism, implementation, and application to benzenedithiol, *J. Chem. Phys.,* **2007,** 126, 174101.

(5) Datta, S., *Electronic Transport in Mesoscopic Systems*. Cambridge University Press: 1995.

(6) Venkataraman, L.; Klare, J. E.; Nuckolls, C.; Hybertsen, M. S.; Steigerwald, M. L., Dependence of single-molecule junction conductance on molecular conformation, *Nature,* **2006,** 442, 904–907.

(7) Park, Y. S.; Widawsky, J. R.; Kamenetska, M.; Steigerwald, M. L.; Hybertsen, M. S.; Nuckolls, C.; Venkataraman, L., Frustrated Rotations in Single-Molecule Junctions, *J. Am. Chem. Soc.,* **2009,** 131, 10820–10821.
